# Hereditary Angioedema in Pregnancy Complicated by Upper Extremity Deep Venous Thrombosis: A Case Report

**DOI:** 10.7759/cureus.68641

**Published:** 2024-09-04

**Authors:** Devon H Newton, Alexandria L Betit, Roshni B Patel, Louis DiValentin

**Affiliations:** 1 Medical Student, Alabama College of Osteopathic Medicine, Dothan, USA; 2 Internal Medicine, Regional Medical Center, Anniston, USA

**Keywords:** central venous catheter, deep venous thromboembolism, c1-inh, pregnancy, hereditary angioedema

## Abstract

Hereditary angioedema (HAE) is a rare disorder that causes episodes of angioedema due to a mutation in the C1 esterase inhibitor gene (C1-INH). Complications of HAE include intestinal obstruction, asphyxiation, and venous thromboembolism (VTE). In this case, we report a 34-year-old G4P2011 female with HAE at 24 weeks gestation presenting with acute right upper extremity pain and swelling following a peripherally inserted central catheter (PICC) line for HAE treatment infusion, revealing a right upper extremity VTE. Early treatment with Lovenox, PICC line removal, and continuation of HAE therapy via peripheral IV infusion resolved and prevented further angioedema and subsequent VTEs during this patient’s pregnancy. This case serves as an example of effective management of HAE complications during pregnancy and supports peripheral IV line usage over PICC lines for medication infusions in pregnant patients with HAE. The overall purpose of this case report is to improve safety outcomes for pregnant patients with HAE by mitigating the risks of PICC line usage and to highlight the significance of VTE inclusion within the differential diagnosis in this population.

## Introduction

Hereditary angioedema (HAE) is a rare autosomal dominant disorder caused by a mutation in the C1 esterase inhibitor gene (C1-INH), predisposing affected individuals towards episodes of angioedema during times of acute stress [1]. Symptoms of HAE include cutaneous, gastrointestinal, and airway angioedema leading to complications such as small intestinal obstruction and asphyxiation [2]. Additionally, research suggests that individuals with HAE are at a significantly elevated risk for venous thromboembolism (VTE) compared to the general population [3]. Episodes of HAE are often unpredictable and severe, and without effective treatment, mortality rates exceed 30% [2]. HAE is thought to affect 1 per 50,000 people in the general population without significant differences in sex or ethnicity [4]. Although males and females are thought to be equally affected by HAE, females may be at an increased risk for more severe cases of angioedema due to the coagulative effects of estrogen [2].

Furthermore, the complex and changing nature of hormones during pregnancy puts patients with HAE at a variable and unpredictable risk of acute attacks which must be carefully monitored and managed by their medical provider [2]. This case report illustrates a 34-year-old G4P2011 female patient at 24 weeks gestation with HAE who presented with an acute peripherally inserted central catheter (PICC) line malfunction and upper extremity VTE treated with Lovenox protocol and PICC line removal. Replacement of the PICC line with a peripheral IV for HAE medication administration resolved and prevented further angioedema and VTEs during this patient’s pregnancy and delivery, supporting the use of peripheral IV lines for primary medical management. Given the inherent hypercoagulable state of both HAE and pregnancy, physicians should have a higher index of suspicion for VTE upon patient presentation and include thrombotic complications in the differential diagnosis.

## Case presentation

A 34-year-old G4P2011 female patient at 24 weeks gestation with HAE presented to the emergency department (ED) complaining of PICC line leakage. After flushing the line and ensuring proper functioning, the patient was discharged home to follow up with home health for medication infusions. No labs were obtained at that time. 

The next day, the patient returned to the ED with two to three days of worsening right upper extremity erythema with pain radiating to the right axilla, and serous leakage from a PICC line on the right arm. She had a past medical history significant for fibromyalgia, arthritis, opioid use disorder on Suboxone, and prior VTE six years ago involving bilateral subclavian veins and proximal right superior vena cava, currently on heparin subcutaneously (SQ). Additionally, the patient was originally taking Haegarda SQ and Firazyr SQ for HAE management. During this pregnancy, she was switched from Firazyr SQ to Ruconest as Firazyr is not preferred for pregnancy. As Ruconest is given intravenously (IV), a PICC line was inserted for infusions ten days prior to this ED evaluation. The patient stated that her last Ruconest infusion was two days before this ED visit. After the infusion, she developed erythema and pain in the right upper arm and described the return of serous leakage from the PICC line, thereby prompting ED evaluation. She admitted to similar symptoms with a previous left upper extremity VTE six years prior. She denied chest pain, shortness of breath, vaginal bleeding, vaginal discharge, and abdominal pain. The patient denied any pertinent family history. Her social history was significant for a five-pack-year smoking history and daily vaping.

**Figure 1:**
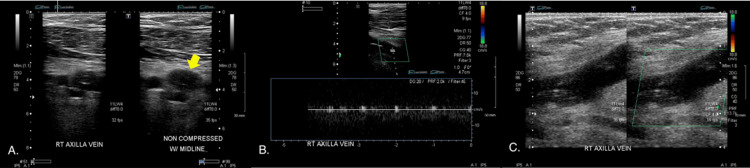
Right upper extremity venous doppler duplex ultrasound of thrombus in right axillary vein Compression (1A), augmentation (1B), and spectral and color Doppler (1C) techniques show non-compressibility (yellow arrow), decreased venous flow (blue box), and absent venous flow (green box) in the axillary vein, respectively.

Physical examination revealed an afebrile, gravid, obese (BMI 34.5) female who appeared uncomfortable and was exhibiting no obvious signs of distress. There was mild edema and induration near the PICC line insertion site on the patient’s right upper arm and tenderness in the medial right upper arm that extend to the right axilla. Complete blood count (CBC) included a leukocytosis of 13.4, neutrophilia of 10.2, and monocytosis of 0.9. Others including comprehensive metabolic panel (CMP), prothrombin time (PT), activated partial thromboplastin time (APTT), international normalized ratio (INR), urine drug screen, and the remainder of CBC were within normal limits. Venous Doppler duplex ultrasound of the right upper extremity showed an occlusive thrombus of the right subclavian, brachial, and axillary veins (Figure 1). There was no thrombus present in the internal jugular vein or cephalic vein.

The patient was subsequently admitted for acute VTE in the setting of PICC line malfunction and chronic HAE. The patient’s PICC line was removed. Home heparin treatment was held. A Lovenox protocol, 1 mg/kg SQ every 12 hours, was initiated. Home HAE medications were continued through a peripheral IV line.

The patient was discharged home on day six of hospitalization with the resolution of her right arm symptoms. Upon discharge, her home heparin treatment was reinitiated, and per cardiology recommendations, the patient was scheduled for placement of an additional PICC line in the left arm within two weeks. Due to patient nonadherence, PICC line placement was delayed to two months post-discharge and ultimately failed due to incompetent veins. Therefore, the patient continued to receive her Ruconest infusions via peripheral IV. The remainder of the patient's pregnancy proceeded without any known complications. Three months after this admission, the patient delivered at 39 1/7 weeks gestation via C-section due to umbilical cord prolapse and fetal decelerations. Delivery was uncomplicated.

## Discussion

Patients with HAE are at greater risk of experiencing acute attacks of angioedema spanning various organ systems [2]. Cutaneous angioedema is the most common symptom of HAE, affecting the face, extremities, and urogenital region [2]. Gastrointestinal angioedema can cause severe abdominal pain and swelling, leading to nausea, vomiting, and diarrhea [2]. Airway angioedema is the least common symptom of HAE but affects at least 50% of patients and results in asphyxiation if not treated [2]. Additional complications of HAE include small intestinal obstruction and VTE [2]. Untreated episodes of angioedema typically last for three to five days [2]. The frequency of attacks varies from weekly to less than once a year [2]. This case highlights the management of acute HAE complications in a pregnant patient, encourages a high index of suspicion for VTE in pregnant patients with HAE, and supports the use of peripheral IV lines instead of PICC lines in patients with an elevated risk for thrombosis.

While males and females are equally affected by HAE, females are predisposed to more severe attacks of angioedema due to the thrombotic effects of estrogen [2]. These attacks become more unpredictable in pregnancy due to the ever-changing hormonal landscape of a gravid individual, making it crucial to carefully mitigate and manage potential HAE complications [2]. Current literature suggests that the frequency of acute HAE attacks increases by 83% in pregnancy [2]. Typical management of HAE includes acute and chronic treatments with C1-INH, kallikrein inhibitors, and receptor antagonists which may be administered via oral route, SQ, or IV infusions [1].

In a pregnant patient, management of HAE must change due to certain medication side effects. Icatibant, known by the brand name Firazyr, is a treatment for acute attacks of HAE and is not preferred for use in pregnancy due to documented preterm birth and miscarriages in animal studies [2]. Drugs currently indicated for the treatment of acute attacks of HAE in pregnancy include plasma-derived C1-INH (Berinert) and Recombinant Human C1-INH (Ruconest) [2]. Medications indicated for long-term prophylaxis of HAE in pregnancy include plasma-derived C1-INH (Cinryze, Haegarda), lanadelumab (Takhzyro), berotralstat (Orladeyo), androgens (danazol), and antifibrinolytics (tranexamic acid) [2].

Most HAE medications are given orally and SQ. However, some medications such as Ruconest, can only be administered IV. IV infusions may be given through a PICC line instead of a peripheral IV due to patient preference, increased comfort, and line durability [5]. However, PICC lines have shown an increased association with upper extremity VTEs [5]. As the use of central venous catheters and PICC lines increases, so does the incidence of upper extremity deep venous thrombosis (DVT) [6]. Upper extremity DVTs constitute 5-10% of all DVTs with an incidence of 14-23% in patients with central venous catheters [6]. Clinical presentation may include standard limb swelling and asymmetry but may be asymptomatic [6]. The most serious complication of an upper extremity DVT is a pulmonary embolism [6]. Therefore, a high clinical index of suspicion for an upper extremity DVT should be present on the differential diagnosis in patients with a central venous catheter or PICC line malfunction, especially in high-risk populations.

Due to the increased risk of thrombotic events in pregnancy and PICC line use, compounded by the inherent risk of VTE in HAE, it is imperative to consider using peripheral IV lines for HAE medication administration instead of PICC lines [2,3,6]. Peripheral IV lines have been associated with a higher safety profile, easier placement, and decreased pain compared to PICC lines [7]. While peripheral IV lines may cause discomfort and complicate the ability to receive home medication infusions, they should be considered when the risks of using a PICC line do not outweigh the benefits, such as in the patient presented in this case. This case presentation illustrates successful medical management utilizing peripheral IV lines instead of a PICC line without additional adverse events or complications. It supports peripheral IV line usage over PICC lines for medication infusions in pregnant patients with HAE. As a result, one goal is to improve safety outcomes for pregnant patients with HAE by mitigating the risks of PICC line usage within this population.

## Conclusions

HAE is an inheritable disorder that causes acute episodes of angioedema that can lead to serious complications such as VTE. Few cases have been reported on the management of HAE complications in pregnant patients. This case supports the importance of including VTE in the differential diagnosis for patients with HAE, showcases the effective management of an acute VTE in a gravid patient with HAE, and examines the benefits of using peripheral IV lines for HAE medication infusions instead of PICC lines in patients with a high risk for thrombosis.
